# Effects of Red and Blue Light on Steady-State and Dynamic Photosynthesis of Cucumber Seedlings

**DOI:** 10.3390/plants15193021

**Published:** 2026-10-02

**Authors:** Tengqi Wang, Qiying Sun, Xinying Liu, Kai Ma, Ce Song, Yaliang Xu, Binbin Liu, Qingming Li

**Affiliations:** 1 Institute of Vegetables, Henan Academy of Agricultural Sciences, Zhengzhou 450002, China; wang_tengqi@163.com (T.W.);; 2 Institute of Urban Agriculture, Chinese Academy of Agricultural Sciences, Chengdu 610213, China; 3 National Chengdu Agricultural Science and Technology Center, Chengdu 610213, China; sunqiying@caas.cn (Q.S.);; 4 College of Food and Biological Engineering, Chengdu University, Chengdu 610106, China

**Keywords:** red/blue light, cucumber (*Cucumis sativus* L.) seedlings, dynamic photosynthesis, chlorophyll fluorescence, Calvin cycle

## Abstract

Our previous study revealed a significant effect of red and blue light on steady-state and dynamic photosynthesis in cucumber seedlings. To further investigate the mechanism by which red and blue light affect photosynthesis, cucumber (*Cucumis sativus* L. cv. Jinyou 365) seedlings were exposed to white light (W), monochromatic red light (R), monochromatic blue light (B), and mixed red–blue light with red:blue photon flux ratios of 9:1 (9R1B), 7:3 (7R3B), 5:5 (5R5B), 3:7 (3R7B), and 1:9 (1R9B). We measured photosynthetic pigment contents, induction curves of photosynthetic gas exchange and chlorophyll fluorescence parameters during low-to-high-light transition, as well as the activities and gene expression of key Calvin cycle enzymes. The results showed that B yielded higher photosynthetic pigment contents than R, although both remained lower than W. Mixed red–blue light with a blue-light ratio above 50% produced significantly higher pigment contents than W. Under low light, blue light exhibited negligible effects on photosynthesis. In contrast, under high light, 7R3B, 5R5B, 3R7B and 1R9B elevated net photosynthetic rate (A) and stomatal conductance (Gs). Among these, 7R3B, 5R5B and 3R7B also accelerated photoinduction, as reflected by a shorter time to reach 90% steady-state net photosynthetic rate (T_90%A_). Chlorophyll fluorescence parameters changed rapidly and attained a steady state within 5 min during light induction. During photoinduction, biochemical limitation was severe in the early stage but declined rapidly, while stomatal limitation appeared in mid-induction and persisted into the late stage; photochemical limitation remained minor, and the severity of these limitations was modified by light quality. Red and blue light exerted negligible effects on chlorophyll fluorescence parameters under low light. However, blue light enhanced the relative electron transport rate of PSII under high light by elevating the fraction of open PSII centers. Furthermore, blue light significantly increased the activities and gene expression of key Calvin cycle enzymes. Correlation analysis revealed that higher chlorophyll contents were positively associated with A and Gs under high light but showed no significant correlation with photoinduction. Higher A and Gs under both low- and high-light intensities were associated with faster photoinduction.

## 1. Introduction

Photosynthesis is an important physiological process whereby green plants absorb carbon dioxide (CO_2_), release oxygen (O_2_), and synthesize organic compounds under light. It is the fundamental metabolic reaction sustaining plant growth and development. Optimizing photosynthetic efficiency is an effective strategy to promote plant growth and improve crop yield [1,2]. Light quality, a critical component of the light environment, serves not only as an energy source for photosynthesis but also as a key signal to modulate plant morphogenesis and carbon assimilation [3,4]. Since red and blue light fall within the peak absorption bands of chlorophyll, numerous studies have investigated their effects on plant photosynthesis [5,6]. It has been demonstrated that red and blue light can affect photosynthesis via two pathways. One involves the modulation of photosynthetic apparatus development, including chlorophyll biosynthesis, chloroplast development, and photosystem biogenesis. The other exerts regulatory effects on photosynthesis, such as modulating stomatal conductance, photosystem activity, and the activities of key Calvin cycle enzymes [7,8]. Beyond driving photosynthesis, red- and blue-light-induced morphophysiological responses are governed by distinct photoreceptor families. Phytochromes (PHY family) perceive red and far-red light, while cryptochromes (CRY1, CRY2) and phototropins (PHOT1, PHOT2) function as major blue-light receptors. Upon light perception, these photoreceptors initiate downstream signaling cascades to regulate key traits, including hypocotyl elongation, leaf development, and the vegetative-to-reproductive phase transition in model and horticultural crops [9,10]. However, physiological responses to red and blue light are highly species-dependent and can differ across developmental stages of the same plant. In Arabidopsis, blue light accelerates floral transition and red light delays flowering, but tomato shows no such effects [10]. In tomato, blue light benefits seedling vegetative growth by preventing excessive hypocotyl elongation [5]. However, when applied after flowering, monochromatic blue light reduces fruit set, even though it produces fruits with better quality [11]. In summary, blue light supports key vegetative growth processes, and red light participates in the regulation of flowering and fruit production. Notably, such responses do not hold true for all plant species or across all growth phases. Monochromatic red or blue light is inadequate to sustain normal plant growth. For example, previous studies have shown that cucumber and tomato seedlings cultivated under monochromatic red or blue light exhibit excessive hypocotyl elongation compared with white light, accompanied by suppressed photosynthesis and plant growth [12,13]. Compared with red light, blue light plays a more critical role in maintaining plant photosynthetic performance. Monochromatic red light triggers red-light syndrome in cucumber seedlings, which results in thin, malformed leaves with reduced chlorophyll content and aberrant chloroplast ultrastructure, thereby severely depressing photosynthesis [14]. However, red-light syndrome can be completely alleviated by supplemental low-intensity blue light [15,16]. Mixed red and blue light is favorable for the growth and photosynthetic performance of most plants, and plant species exhibit distinct responses to alterations in red-to-blue-light ratios [17,18]. For example, previous studies have shown that increasing the blue-light ratio can suppress excessive hypocotyl elongation and improve the maximum photosynthetic capacity of cucumber seedlings, whereas adjusting the blue-light ratio exerts a minor effect on tomato seedlings [19,20]. Therefore, the responses of plant growth and photosynthesis to red and blue light should be further explored to identify the optimal red-to-blue light ratio for individual plant species.

Photosynthetic capacity in plants can be divided into two components: steady-state photosynthesis and dynamic photosynthesis. Most previous studies have focused on steady-state photosynthesis, which estimates photosynthetic capacity by measuring physiological parameters under fixed environmental conditions [21,22]. Relevant parameters include the maximum net photosynthetic rate (A_max_), the maximum stomatal conductance (Gs_max_), and intrinsic water-use efficiency (iWUE) measured under saturating light. These parameters have attracted widespread attention in photosynthesis-related studies; improving A_max_ and iWUE has long been considered an effective strategy to improve crop yield [23]. However, with advances in photosynthesis research, an increasing number of studies have demonstrated that steady-state photosynthetic capacity cannot fully reflect the actual photosynthetic performance of crops under field conditions [24,25]. Under field conditions, most environmental factors, including light intensity, temperature and humidity, fluctuate continuously, thereby altering plant photosynthetic performance. Dynamic photosynthetic capacity describes the capacity of plants to adjust photosynthetic carbon assimilation under fluctuating environments, thereby enhancing CO_2_ assimilation efficiency and iWUE [26,27]. As a major environmental determinant of photosynthesis, fluctuations in light intensity exert direct effects on carbon assimilation. Under natural conditions, the light intensity incident on plant leaves varies continuously owing to diurnal changes, cloud cover, and mutual leaf shading [28,29]. Previous studies have demonstrated that the pattern, frequency, and duration of light fluctuations exert a substantial influence on plant photosynthesis, growth, and development [30,31]. In general, plants can alleviate photodamage by adjusting light absorption and utilization under short-term fluctuating light, but prolonged and large-amplitude light fluctuations will induce photodamage due to the limited regulatory capacity of plants [32,33]. In the short term, fluctuating light affects photosynthesis in two ways: when light intensity rises rapidly, the net photosynthetic rate (A) increases slowly, which leads to incomplete utilization of light and CO_2_, thereby causing carbon loss and even photodamage. In contrast, when light intensity drops, A decreases rapidly whereas Gs declines slowly, which results in excessive transpirational water loss, thereby reducing iWUE [34,35]. Optimizing the dynamic photosynthetic capacity of plants is an effective strategy to improve light use efficiency, CO_2_ use efficiency and water-use efficiency, thereby promoting plant growth and increasing crop yield [36,37].

Recent studies have demonstrated that dynamic photosynthetic capacity can markedly affect crop yield, whereas the key factors limiting this capacity differ across plant species and environmental conditions [38,39]. One previous study identified stomatal limitation as the key factor for dynamic photosynthetic capacity in cucumber, while other studies reported that biochemical processes are the main limiting factors in rice [16,40]. These inconsistent results may be attributed to the fact that rice is a monocot grass species possessing dumbbell-shaped guard cells, which open and close more rapidly in response to fluctuating light, thereby alleviating stomatal limitation. Therefore, it is necessary to further explore the responses of different plant species to different fluctuating light regimes to identify the critical limiting factors of dynamic photosynthesis. Cucumber is one of the most widely cultivated commercial vegetables worldwide, and numerous studies have examined how light quality regulates photosynthesis and growth of cucumber to develop new strategies for improving its yield and stress tolerance [41,42]. Our previous study showed that red and blue light regulate not only steady-state photosynthesis but also dynamic photosynthesis of cucumber seedlings, yet the underlying molecular mechanisms remain poorly understood [16]. Accordingly, cucumber seedlings were grown under white light and various red-to-blue-light treatments in this experiment. We measured photosynthetic pigment concentrations, induction curves of photosynthetic gas exchange and chlorophyll fluorescence parameters, and activities of key Calvin cycle enzymes, as well as the expression levels of the corresponding genes. This study aims to identify key factors affecting dynamic photosynthesis of cucumber seedlings, thereby providing a theoretical basis and new perspectives for subsequent photosynthesis-related research.

## 2. Results

### 2.1. Effects of Red and Blue Light on Leaf Photosynthetic Pigment Contents of Cucumber Seedlings

**Figure 1:**
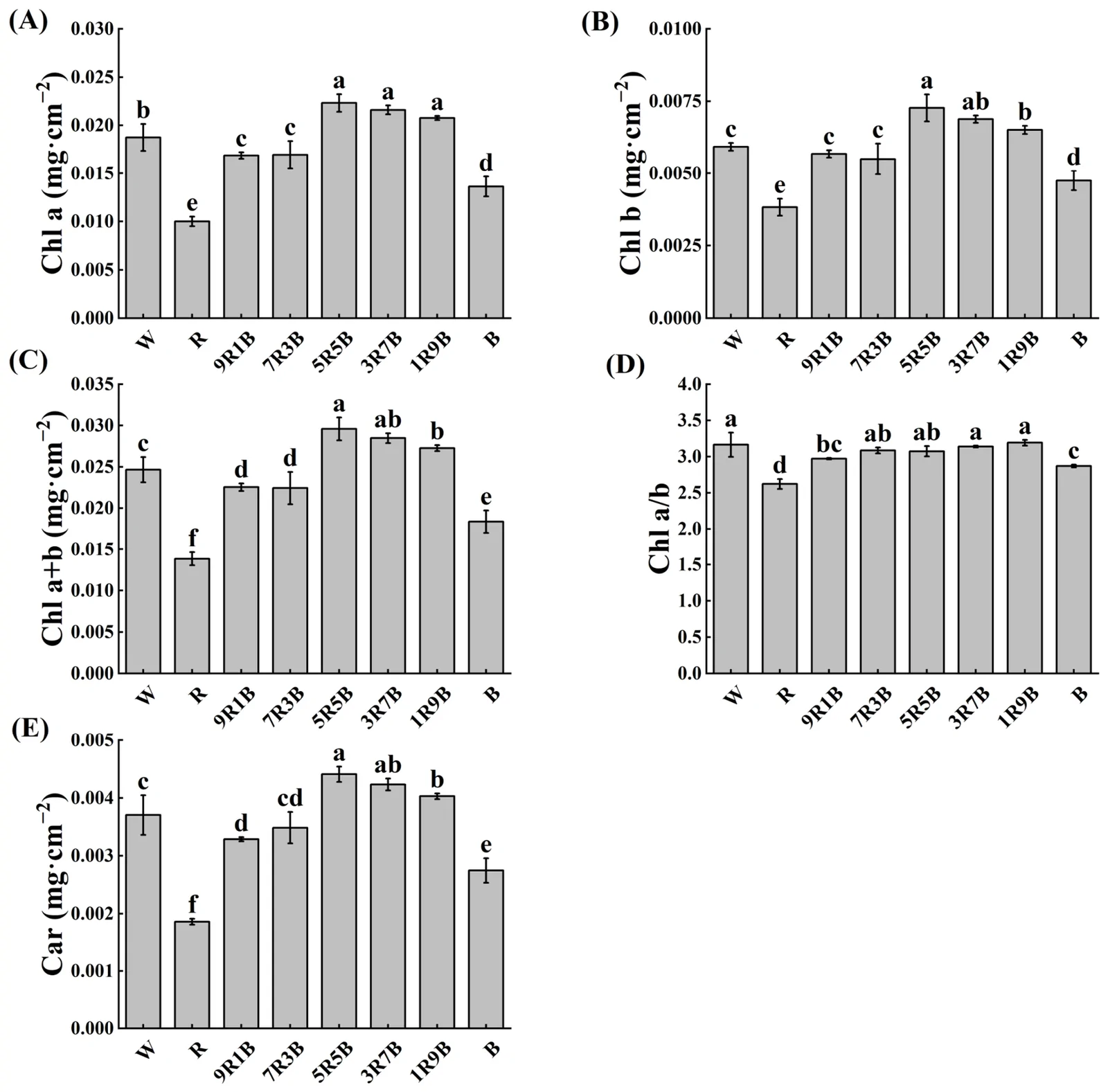
Effects of red and blue light on leaf photosynthetic pigment contents of cucumber seedlings. (**A**) Chlorophyll a content per leaf area (Chl a), (**B**) chlorophyll b content per leaf area (Chl b), (**C**) total chlorophyll content per leaf area (Chl a + b), (**D**) ratio of chlorophyll a content to chlorophyll b content (Chl a/b), (**E**) carotenoid content per leaf area (Car). White light (W), monochromatic red light (R), 90% red and 10% blue mixed light (9R1B), 70% red and 30% blue mixed light (7R3B), 50% red and 50% blue mixed light (5R5B), 30% red and 70% blue mixed light (3R7B), 10% red and 90% blue mixed light (1R9B), and monochromatic blue light (B). Different letters indicate significant differences among treatments (*p* < 0.05).

Photosynthetic pigment contents per leaf area under different treatments are shown in Figure 1. Compared with W, both R and B significantly reduced Chl a, Chl b, Chl a + b, Chl a/b and Car, and the reduction was more obvious under R. Compared with W, Chl a/b was significantly reduced under 9R1B and showed no significant differences from W under other mixed red–blue-light treatments. Under mixed red and blue-light treatments, increasing the blue-light ratio could increase leaf photosynthetic pigment contents. The levels of Chl a, Chl b, Chl a + b and Car under 9R1B and 7R3B were comparable to or lower than those under W, whereas these photosynthetic pigment contents increased significantly under 5R5B, 3R7B and 1R9B, peaking at 5R5B. Relative to 5R5B, Chl b, Chl a + b and Car declined markedly under 1R9B but remained significantly higher than those under W. In brief, blue light promoted photosynthetic pigment accumulation under mixed red–blue light, and this positive effect was more pronounced when the blue-light ratio reached 50%. However, the positive effect of blue light on photosynthetic pigment contents per fresh weight was not obvious (Figure S1).

### 2.2. Variations in Photosynthetic Gas-Exchange Parameters During Light Induction

**Figure 2:**
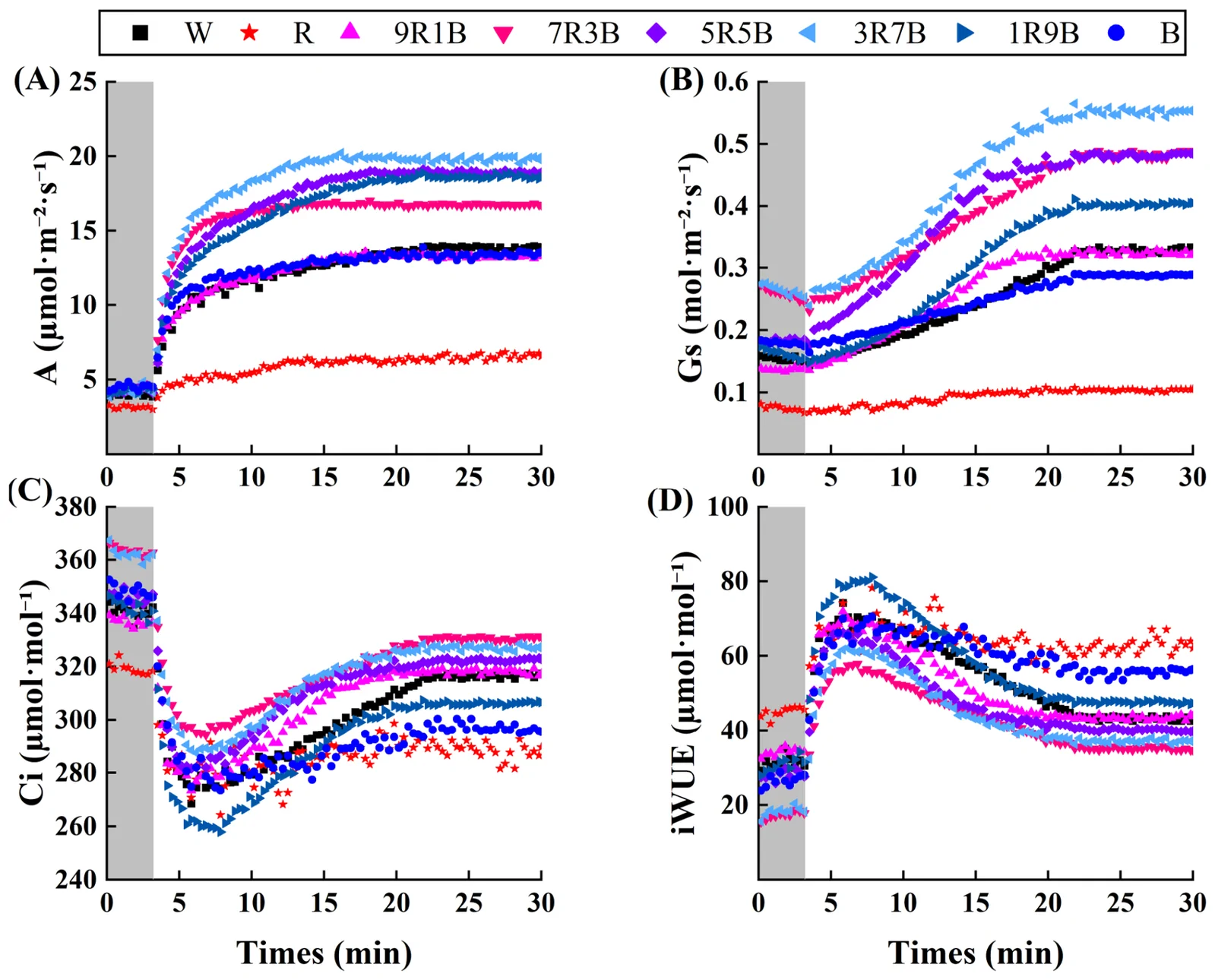
Variations in photosynthetic gas-exchange parameters during light induction. (**A**) Net photosynthetic rate (A), (**B**) stomatal conductance (Gs), (**C**) intercellular carbon dioxide concentration (Ci), (**D**) intrinsic water-use efficiency (iWUE). The gray area represents a light intensity of 100 μmol·m^−2^·s^−1^, and the white area represents a light intensity of 1500 μmol·m^−2^·s^−1^. Light intensity was increased at 3 min, and data were recorded at 10 s intervals.

As shown in Figure 2, to investigate the effects of red and blue light on dynamic photosynthesis of cucumber seedlings, we measured the light-induction curves of net photosynthetic rate (A), stomatal conductance (Gs), intercellular CO_2_ concentration (Ci), and intrinsic water-use efficiency (iWUE). Light intensity was increased from 100 μmol·m^−2^·s^−1^ to 1500 μmol·m^−2^·s^−1^ during the induction process. The results showed that during light induction, A increased slightly under R, whereas it rose rapidly and stabilized within 10–15 min under all other treatments. Gs changed slightly under R, while it increased slowly under other treatments and peaked at 20–25 min. Ci decreased rapidly to its minimum at the early stage of light induction, followed by a slow increase until stabilization at 20–25 min. iWUE rose rapidly to the maximum at the initial stage, followed by a slow decline and stabilization within 20–25 min.

### 2.3. Effects of Red and Blue Light on Steady-State Photosynthetic Gas-Exchange Parameters of Cucumber Seedlings

As shown in Table 1, compared with W, initial net photosynthetic rate before light induction (A_i_) varied slightly under different treatments. Although A_i_ reached the minimum value under R, no significant difference was found between R and W. The steady-state net photosynthetic rate after light induction (A_f_) was significantly affected by red and blue light. Compared with W, A_f_ decreased significantly by 51.46% under R but varied slightly under B. Under mixed red and blue-light treatments, A_f_ showed an upward trend with the increase in blue-light ratio. A_f_ increased significantly by 20.38%, 35.23%, 43.03% and 32.95% under 7R3B, 5R5B, 3R7B and 1R9B, respectively, compared with W. A_f_ reached the highest value under 3R7B and the lowest value under R. Compared with W, the initial stomatal conductance before light induction (Gs_i_) was significantly decreased under R, but it varied slightly under other treatments. The steady-state stomatal conductance after light induction (Gs_f_) reached the lowest value under R and was significantly reduced by 64.60% relative to W. Under mixed red and blue-light treatments, Gs_f_ increased with the increase in blue-light ratio and reached the highest value under 3R7B. Compared with W, the total increment in net photosynthetic rate during light induction (A_f_ − A_i_) decreased significantly under R, but changed slightly under B. Under mixed red and blue-light treatments, A_f_ − A_i_ increased with the increase in blue-light ratio and reached the highest value under 3R7B. The variation trend of A_f_ − A_i_ was similar to that of A_f_. Compared with W, the total increment in stomatal conductance during light induction (Gs_f_ − Gs_i_) changed slightly under R and B. Under mixed red and blue-light treatments, Gs_f_ − Gs_i_ increased with the increase in blue-light ratio and reached the highest value under 3R7B. In summary, blue light could significantly increase A and Gs under high-light intensity (1500 μmol·m^−2^·s^−1^). However, the positive effects of blue light on A and Gs were not obvious under low-light intensity (100 μmol·m^−2^·s^−1^).

### 2.4. Effects of Red and Blue Light on Dynamic Photosynthetic Gas-Exchange Parameters of Cucumber Seedlings

As shown in Table 2, compared with W, time to reach 50% steady-state net photosynthetic rate (T_50%A_) was significantly increased under R, but varied slightly under other treatments. Time to reach 90% steady-state net photosynthetic rate (T_90%A_) changed slightly under R and B, but was significantly decreased by 51.53%, 42.71% and 50.85% under 7R3B, 5R5B and 3R7B, respectively, relative to W. T_90%A_ reached the lowest value under 7R3B and reached the highest value under R. Time to reach 50% steady-state stomatal conductance (T_50%Gs_) was significantly reduced under R, 7R3B and 5R5B, and reached the lowest value under 5R5B. Compared with W, time to reach 90% steady-state stomatal conductance (T_90%Gs_) was significantly reduced under 9R1B, but showed no significant differences among the other treatments. Lag time for stomatal conductance response (λ) decreased significantly under R and increased significantly under B. Under mixed red and blue-light treatments, λ decreased significantly by 61.13%, 63.13%, 74.03% and 57.01% under 7R3B, 5R5B, 3R7B and 1R9B, respectively, relative to W. λ reached the lowest value under 3R7B and the highest value under B. Compared with W, the maximum increase rate of stomatal conductance (Sl_max_) decreased significantly and reached the lowest value under R, whereas it increased significantly and reached the highest value under 1R9B.

### 2.5. Variation in Chlorophyll Fluorescence Parameters During Light Induction

**Figure 3:**
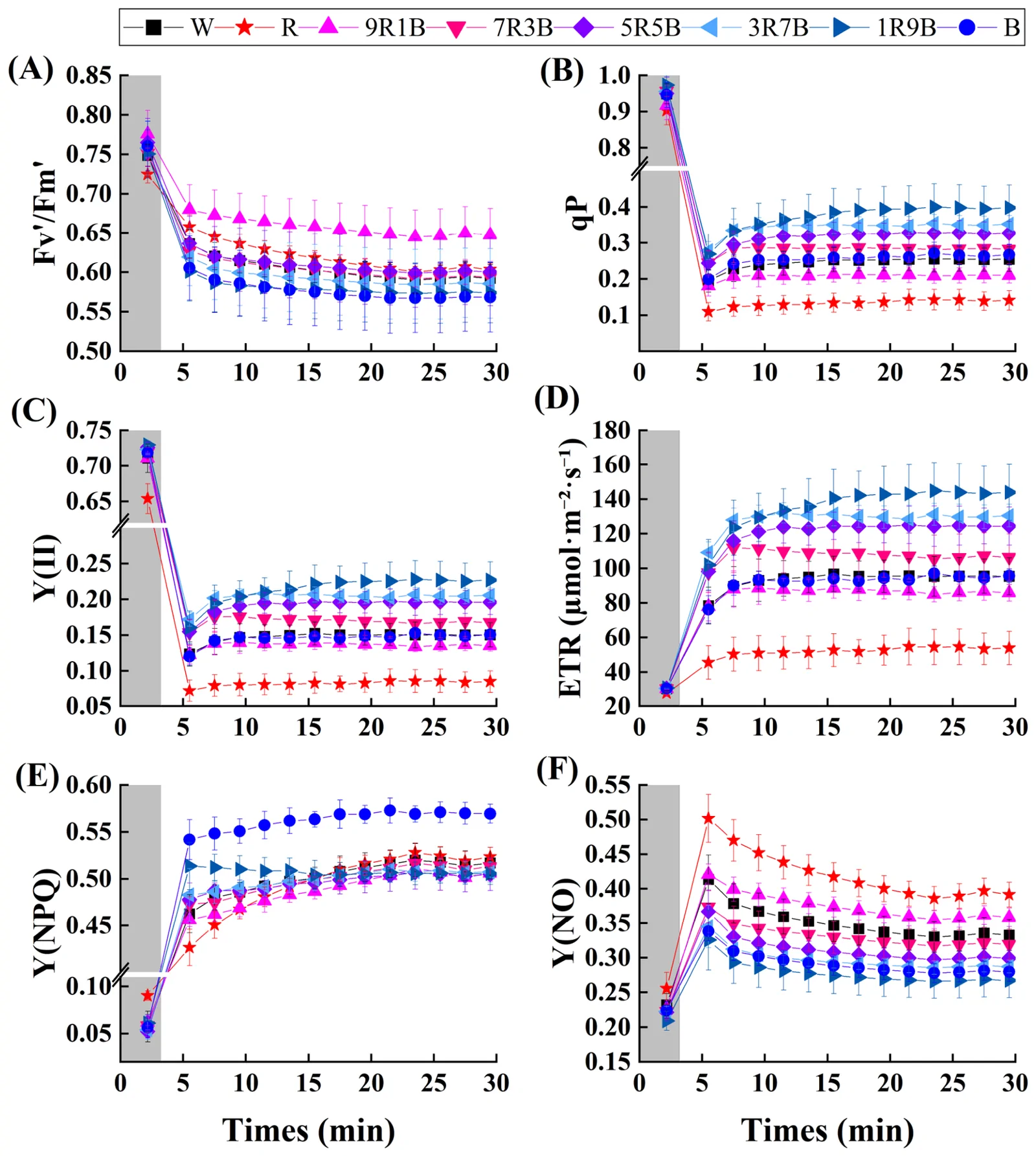
Variation in chlorophyll fluorescence parameters during light induction. (**A**) Maximum photochemical efficiency of PSII under light adaptation (Fv’/Fm’), (**B**) photochemical quenching coefficient of PSII (qP), (**C**) actual photochemical quantum yield of PSII (Y(II)), (**D**) relative electron transport rate of PSII (ETR), (**E**) quantum yield of regulated non-photochemical energy dissipation of PSII (Y(NPQ)), (**F**) quantum yield of non-regulated non-photochemical energy dissipation of PSII (Y(NO)). The gray area represents a light intensity of 100 μmol·m^−2^·s^−1^, and the white area represents a light intensity of 1500 μmol·m^−2^·s^−1^. Data were recorded at 2 min intervals.

As shown in Figure 3, during light induction, maximum photochemical efficiency of PSII under light adaptation (Fv’/Fm’) decreased rapidly within the first 2 min and then slowly declined to its minimum. During light induction, the photochemical quenching coefficient of PSII (qP) decreased rapidly and reached its minimum at 5 min, followed by a slight increase and subsequent steady state. The variation trend of actual photochemical quantum yield of PSII (Y(II)) was consistent with that of qP, indicating that the fraction of open PSII centers was a key factor affecting its electron transport capacity during light induction. The relative electron transport rate of PSII (ETR) increased rapidly and reached a steady state at 10 min. The quantum yield of regulated non-photochemical energy dissipation of PSII (Y(NPQ)) increased rapidly at the beginning of light induction, and then fluctuated slightly during light induction. Quantum yield of non-regulated non-photochemical energy dissipation of PSII (Y(NO)) rose rapidly to its maximum at 5 min, and then gradually declined to the minimum. These results indicated rapid activation of the photosystems during light induction, implying that the activation of photosynthetic electron transport was not the primary limiting factor for photoinduction.

### 2.6. Effects of Red and Blue Light on Chlorophyll Fluorescence Parameters of Cucumber Seedlings

**Figure 4:**
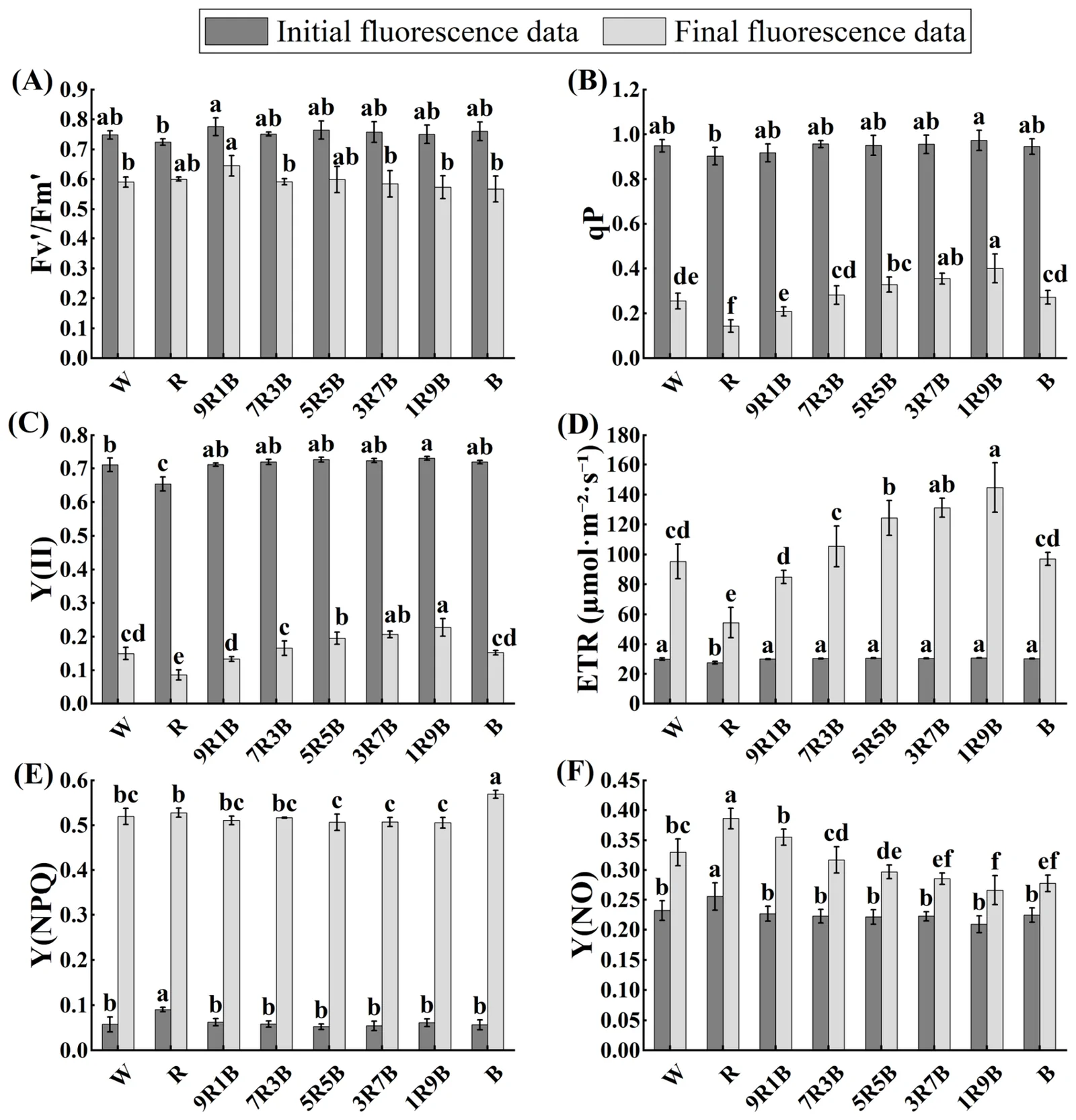
Effects of red and blue light on chlorophyll fluorescence parameters of cucumber seedlings. (**A**) Maximum photochemical efficiency of PSII under light adaptation (Fv’/Fm’), (**B**) photochemical quenching coefficient of PSII (qP), (**C**) actual photochemical quantum yield of PSII (Y(II)), (**D**) relative electron transport rate of PSII (ETR), (**E**) quantum yield of regulated non-photochemical energy dissipation of PSII (Y(NPQ)), (**F**) quantum yield of non-regulated non-photochemical energy dissipation of PSII (Y(NO)). Dark gray columns represent chlorophyll fluorescence parameters under low-light intensity (100 μmol·m^−2^·s^−1^), light gray columns represent chlorophyll fluorescence parameters under high-light intensity (1500 μmol·m^−2^·s^−1^). The difference analysis was individually conducted under two light intensities. Different letters indicate significant differences among treatments (*p* < 0.05).

As shown in Figure 4, under low-light intensity (100 μmol·m^−2^·s^−1^), Y(II) and ETR were significantly decreased under R, whereas Y(NPQ) and Y(NO) were significantly increased. In contrast, other chlorophyll fluorescence parameters showed little variation among treatments. Under high-light intensity (1500 μmol·m^−2^·s^−1^), Fv’/Fm’ was significantly higher under 9R1B relative to W, whereas it showed little variation in the remaining treatments. R significantly decreased qP, Y(II) and ETR, but significantly increased Y(NO). B significantly increased Y(NPQ) and reduced Y(NO), while it had no significant effects on qP, Y(II) and ETR. Under mixed red and blue-light treatments, with increasing blue-light ratio, qP, Y(II) and ETR increased, whereas Y(NO) and Y(NPQ) decreased. Under 1R9B, qP, Y(II) and ETR reached their maximum values, whereas Y(NPQ) and Y(NO) reached their minimum values. These results indicated that blue light could significantly enhance the electron transport and photoprotective capacity of PSII under high-light intensity, whereas this effect was negligible under low-light intensity. The inhibitory effect of red light on the electron transport capacity of PSII was significant under both low and high-light intensities.

### 2.7. Effects of Red and Blue Light on Activities and Gene Expression of Key Calvin Cycle Enzymes of Cucumber Seedlings

**Figure 5:**
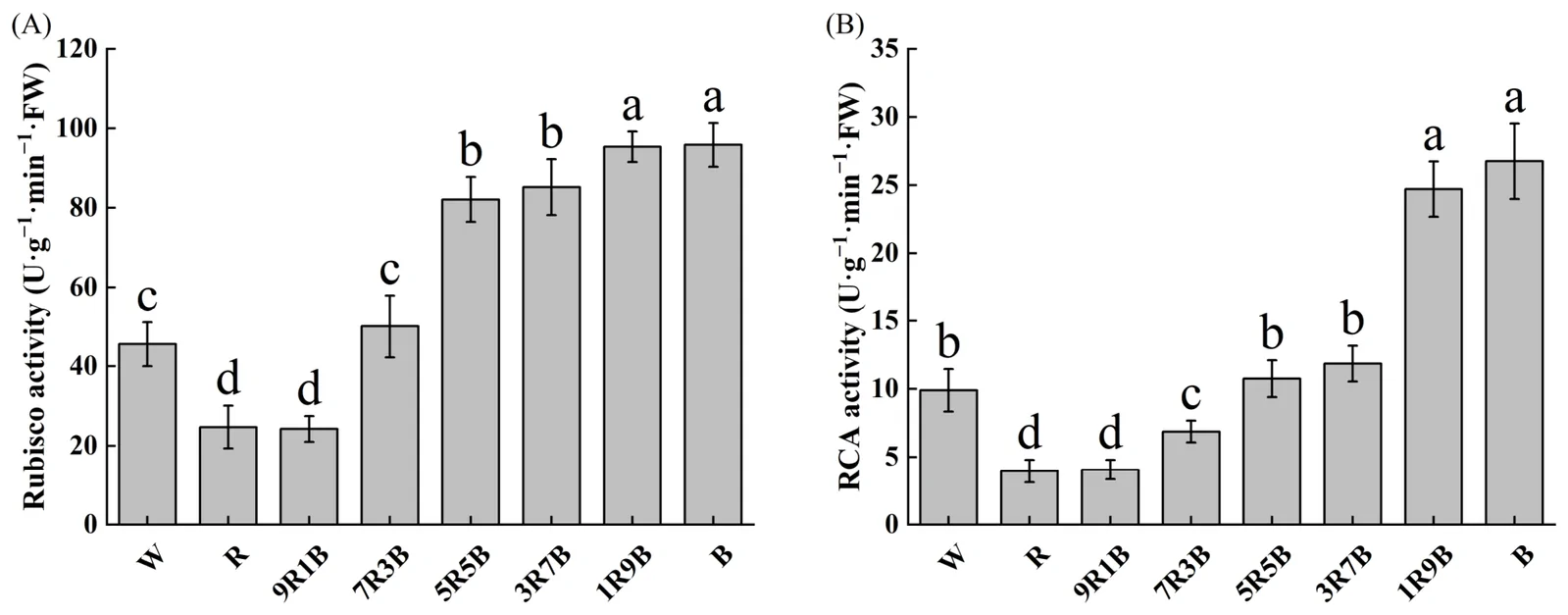
Effects of red and blue light on activities of key Calvin cycle enzymes in cucumber seedlings. (**A**) Ribulose-1,5-bisphosphate carboxylase/oxygenase activity (Rubisco activity), (**B**) Rubisco activase activity (RCA activity). Different letters indicate significant differences among treatments (*p* < 0.05).

As shown in Figure 5, ribulose-1,5-bisphosphate carboxylase/oxygenase activity (Rubisco activity) decreased significantly under R and 9R1B, by 45.73% and 46.75%, respectively, relative to W. Rubisco activity rose gradually with increasing blue-light ratio; it elevated significantly under 5R5B, 3R7B, 1R9B and B, with the maximum value observed under B. The variation trend of Rubisco activase activity (RCA activity) was consistent with that of Rubisco. These results demonstrated that blue light could effectively elevate the activities of Rubisco and RCA.

**Figure 6:**
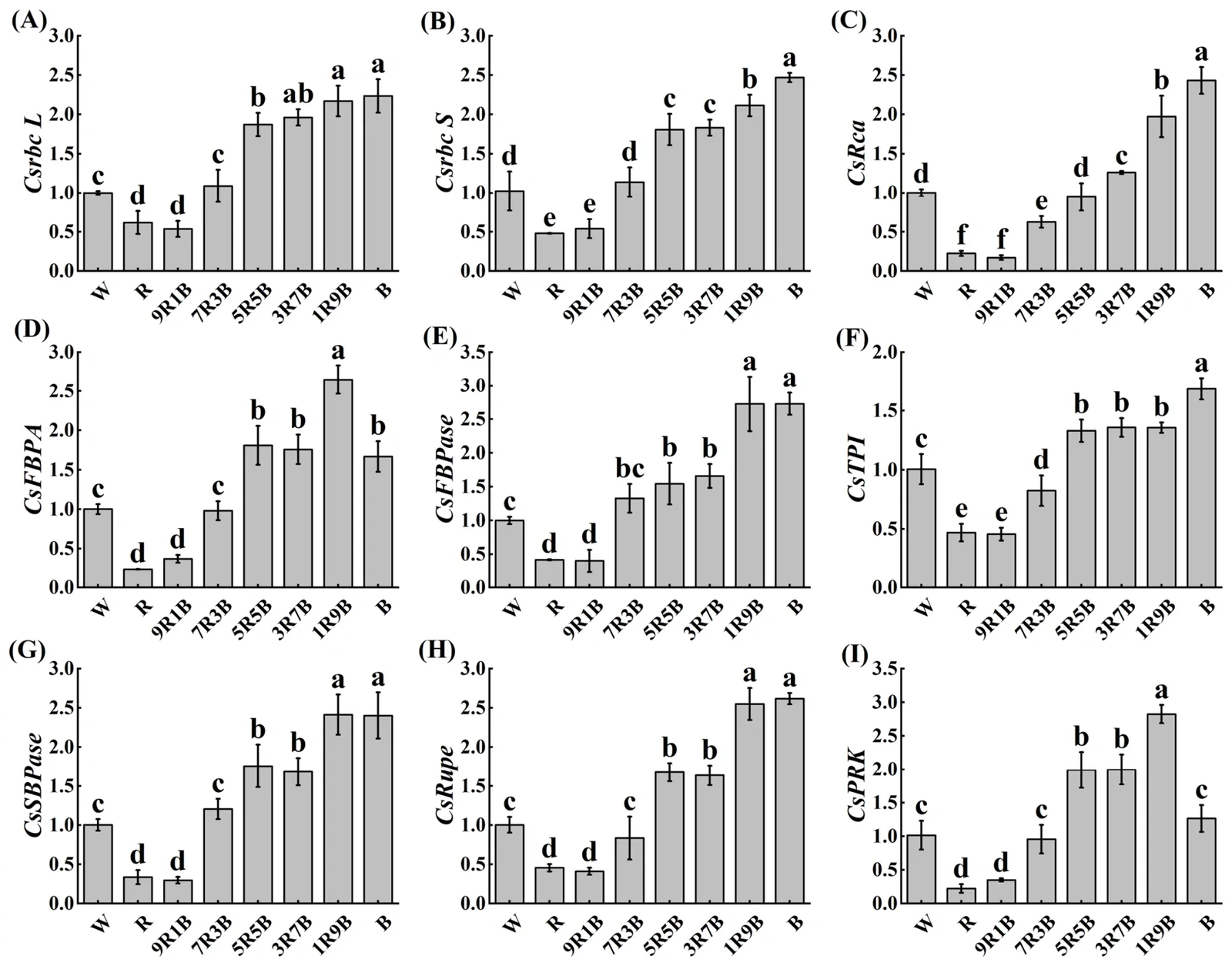
Effects of red and blue light on gene expression of key Calvin cycle enzymes in cucumber seedlings. (**A**) Expression of Rubisco large subunit gene (*CsrbcL*), (**B**) expression of Rubisco small subunit gene (*CsrbcS*), (**C**) expression of Rubisco activase gene (*CsRca*), (**D**) expression of fructose-1,6-bisphosphate aldolase gene (*CsFBPA*), (**E**) expression of fructose-1,6-bisphosphatase gene (*CsFBPase*), (**F**) expression of triose-phosphate isomerase gene (*CsTPI*), (**G**) expression of sedoheptulose-1,7-bisphosphatase gene (*CsSBPase*), (**H**) expression of ribulose-5-phosphate epimerase gene (*CsRupe*), (**I**) expression of phosphoribulokinase gene (*CsPRK*). Different letters indicate significant differences among treatments (*p* < 0.05).

As shown in Figure 6, R and 9R1B significantly reduced the expression of the cucumber Rubisco large subunit gene (*CsrbcL*) by 38.25% and 46.36%, respectively, relative to W. *CsrbcL* increased gradually with an elevated ratio of blue light, peaking under B. The expression patterns of cucumber Rubisco small subunit gene (*CsrbcS*) and Rubisco activase gene (*CsRca*) under different treatments were similar to those of *CsrbcL*. The expression of the cucumber fructose-1,6-bisphosphate aldolase gene (*CsFBPA*) was significantly decreased by 76.37% and 63.12% under R and 9R1B, respectively, relative to W. *CsFBPA* was significantly increased under 5R5B, 3R7B, 1R9B and B, and peaked under 1R9B. The expression of the cucumber fructose-1,6-bisphosphatase gene (*CsFBPase*) was significantly decreased by 58.42% and 59.87% under R and 9R1B, respectively, relative to W, whereas its expression was markedly higher under 5R5B, 3R7B, 1R9B and B. The expression of the cucumber triose-phosphate isomerase gene (*CsTPI*) was significantly decreased by 53.34%, 54.69% and 18.12% under R, 9R1B and 7R3B, respectively, relative to W, whereas its expression was significantly increased under 5R5B, 3R7B, 1R9B and B, with the maximum value observed under B. The expression patterns of the cucumber sedoheptulose-1,7-bisphosphatase gene (*CsSBPase*) and ribulose-5-phosphate epimerase gene (*CsRupe*) under different treatments were similar to those of *CsFBPase*. The expression pattern of the cucumber phosphoribulokinase gene (*CsPRK*) under different treatments was similar to that of *CsFBPA*. These results indicated that blue light significantly upregulated the transcript levels of key Calvin cycle enzyme genes in cucumber.

### 2.8. Correlation Analysis of Key Parameters

**Figure 7:**
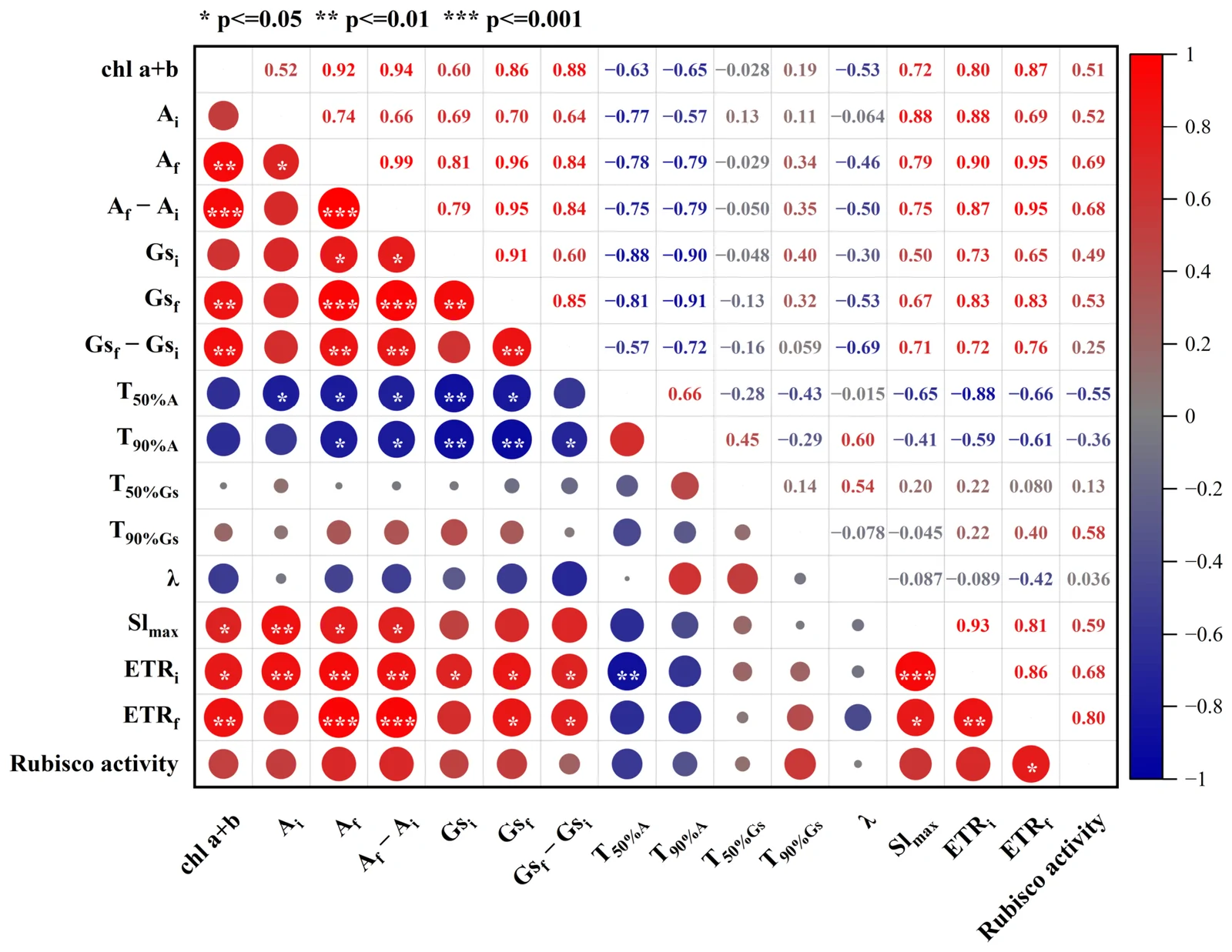
Correlation analysis of key parameters. The color of circles represents the correlation coefficient value, and circle size represents the absolute magnitude of correlation coefficients. Red circles and positive values represent positive correlations, whereas blue circles and negative values represent negative correlations. ‘*’ represents a significant correlation at *p* ≤ 0.05, ‘**’ at *p* ≤ 0.01, and ‘***’ at *p* ≤ 0.001.

As shown in Figure 7, Chl a + b was positively correlated with A_f_, A_f_ − A_i_, Gs_f_, Gs_f_ − Gs_i_, ETR_i_ and ETR_f_, whereas no significant correlation was observed between Chl a + b and A_i_ or Gs_i_. These results indicated that higher chlorophyll content was closely associated with the maximum photosynthetic capacity of cucumber seedlings. A_f_ was positively correlated with Gs_f_, whereas no significant correlation was detected between A_i_ and Gs_i_. These results indicated that A was closely associated with Gs under high light, but such an association was not significant under low light. A_f_ − A_i_ was positively correlated with A_f_, whereas no significant correlation was detected between A_f_ − A_i_ and A_i_. Similar results were also observed for Gs_f_ and Gs_f_ − Gs_i_. For dynamic photosynthesis, T_50%A_ and T_90%A_ were negatively correlated with A_f_, A_f_ − A_i_, Gs_i_, and Gs_f_. In addition, T_50%A_ was negatively correlated with A_i_ and ETR_i_, while T_90%A_ was negatively correlated with Gs_f_ − Gs_i_. These results indicated that higher A and Gs under both low and high-light intensities were closely associated with faster photoinduction. Notably, higher initial A and ETR were associated with a faster early induction phase, whereas a larger Gs_f_ − Gs_i_ was associated with a faster late induction phase. T_50%Gs_, T_90%Gs_ and λ exhibited no significant correlations with the other parameters. Sl_max_ was positively correlated with Chl a + b, A_i_, A_f_, A_f −_ A_i_, ETR_i_ and ETR_f_, whereas no significant correlation was observed between Sl_max_ and other dynamic photosynthetic parameters. Rubisco activity was positively correlated with ETR_f_, but exhibited no significant correlation with the remaining parameters.

## 3. Discussion

### 3.1. Photosynthetic Pigment Contents and Photosynthetic Capacity

Monochromatic blue light yielded higher leaf-area-based photosynthetic pigment contents than monochromatic red light, although both were lower than white light. The promotive effect of blue light on leaf photosynthetic pigment contents was more obvious under mixed red–blue light with a blue-light ratio above 50%, where pigment contents were significantly higher than under white light (Figure 1). This result was consistent with previous studies reporting that blue light could increase the activities of chlorophyll-synthesizing enzymes and the expression levels of relevant functional genes, thereby elevating chlorophyll contents [43,44]. Increasing photosynthetic pigment contents is an important way for blue light to enhance the maximum photosynthetic capacity of plants [18,45]. However, the positive effect of blue light on photosynthetic pigment contents per leaf fresh weight was not obvious (Figure S1). Such inconsistency has also been documented in earlier work: photosynthetic pigment contents per unit leaf area increase with elevated blue-light ratio under mixed red–blue light, whereas pigment contents per leaf dry weight decline [46]. The increase in area-based pigment contents is strongly associated with blue-light-driven rises in leaf mass per unit leaf area (LMA). Our previous study also demonstrated that blue light significantly increased LMA of the first true leaf in cucumber seedlings under mixed red–blue light, which supports this inference [16]. Notably, such a difference may also be related to leaf water content and leaf anatomical structure, and further experiments are needed to explore the mechanisms behind this difference.

For steady-state photosynthesis, compared with red light, blue light could increase A_f_ and Gs_f_, as well as A_f_ − A_i_ and Gs_f_ − Gs_i_; the effect was more obvious under 3R7B, but the positive effect of blue light on A_i_ and Gs_i_ was insignificant (Table 1). These results indicated that the positive effects of blue light on photosynthesis were more obvious under high light rather than low light, as blue light improved photosynthetic performance mainly by elevating maximum stomatal conductance, maximum PSII electron-transport capacity, and maximum capacity for Calvin-cycle-dependent CO_2_ fixation. Under low light, when photosynthetic reactions were not fully activated, photon availability itself became the predominant limiting factor; even improved stomatal, photochemical, and biochemical capacities could not produce promoting effects when photons were insufficient to drive carbon fixation. This finding was consistent with previous studies reporting that blue light improved the maximum photosynthetic capacity of cucumber and eggplant by elevating stomatal conductance, chlorophyll contents and photosystem activities [14,47]. The positive effect of blue light on photosynthesis was not obvious under low light, and the leaf area of cucumber seedlings decreased with increasing blue light ratio [16]. Reduced leaf area and unchanged actual photosynthetic rate can lead to declines in whole-plant photosynthetic capacity and biomass accumulation. Therefore, subsequent photosynthesis-related research should pay more attention to whole-plant photosynthetic performance under diverse growth environments, since the maximum photosynthetic capacity per leaf area cannot fully reflect the actual photosynthetic performance of plants.

In previous studies, we found that blue light could significantly accelerate photoinduction and stomatal opening during the dark-to-high-light transition [16]. In this experiment, by measuring induction curves during the transition from low-to-high light, we found that the induction rates of A and Gs were higher than those measured in our previous study (Table 2), indicating that low-light pre-irradiation could accelerate photoinduction by activating photosynthesis-related reactions and promoting stomatal opening. Similar results have been reported for *Arabidopsis thaliana*. A previous study revealed that increasing stomatal density and stomatal conductance under low light through *ST-OX* overexpression or *EPF1* knockout was an effective strategy to accelerate photoinduction [48]. In addition, a previous study found that light supplementation in the morning was an effective way to promote tomato growth by pre-activating the photosynthetic apparatus and improving stomatal function [49]. In this experiment, the positive effect of blue light on photoinduction was only observed under mixed red and blue light, whereas its influence on stomatal induction was insignificant (Table 2). This result implies that the positive effect of blue light on photoinduction could be weakened by low-light pre-treatment, since A and Gs were partially activated. Under mixed red and blue light, the photoinduction rate peaked under 3R7B but decreased under 1R9B (Table 2), indicating that excessive blue light negatively affected dynamic photosynthesis. Similarly, previous studies have reported that high blue light intensity reduces tomato biomass and fruit yield [50]. In summary, 3R7B yielded the highest steady-state net photosynthetic capacity (A_f_), while multiple mixed-light treatments (7R3B, 5R5B, 3R7B) accelerated photoinduction, indicating that the optimal red-to-blue ratio differs between steady-state and dynamic photosynthesis.

During light induction, biochemical reactions constituted the primary limiting factor for photoinduction at the early stage. Since CO_2_ at the carboxylation site was sufficient for photosynthesis, stomatal limitation remained weak (Figure S2). This finding was consistent with previous studies on rice and soybean, which reported that the operation of the Calvin cycle, particularly Rubisco activation, was a key determinant of light induction [40,51]. Compared with biochemical limitation, stomatal limitation showed a lower maximum value but persisted for a longer time (Figure S2). This result can be explained by the fact that stomata serve as vital structures enabling plants to balance water loss and CO_2_ absorption throughout light induction [52,53]. In this study, the durations of stomatal and biochemical limitations were shorter than those observed during the dark-to-high-light transition, indicating that low-light pre-treatment can activate stomata and photosynthesis-associated biochemical reactions, thereby reducing their limitations on photoinduction [16].

### 3.2. Photosynthetic Electron Transport, Key Calvin Cycle Enzyme Activities and Gene Expression

During light induction, chlorophyll fluorescence parameters reached a stable state rapidly (Figure 3), indicating that the photosynthetic electron transport chain was rapidly activated by high light, and therefore imposed no long-term limitation on photoinduction. This finding differed from those of previous studies, which reported that photosynthetic electron transport could markedly affect photoinduction. For instance, the photoinduction rate in tomato could be enhanced by promoting the activation of this pathway [54,55]. The different results may be because the low-light irradiation before light induction partially activated photosystems, thereby enhancing their adaptability to high light and alleviating the limitation of the photosynthetic electron transport chain on photoinduction. In addition, blue light could enhance the electron transport capacity of PSII under high light by increasing the openness of PSII (Figure 4), indicating that blue light could facilitate the downstream reactions of PSII under high light intensity and thereby boost photosynthetic electron transport. Similarly, previous studies have found that blue light could significantly improve the electron transport capacity of tomato and eggplant [3,47]. Monochromatic blue light significantly increased Y(NPQ) and decreased Y(NO). Under mixed red and blue light, elevating the blue light ratio increased Y(II) but reduced Y(NPQ) and Y(NO) (Figure 4). These results indicated that blue light could optimize the energy partitioning of PSII, enhance light use efficiency and alleviate photodamage, which were consistent with those of previous studies [46,56]. The promotive effect of blue light on photosynthetic electron transport was negligible under low light. In contrast, monochromatic red light obviously inhibited photosynthetic electron transport even under low light (Figure 4). In summary, blue light was indispensable for the normal operation of photosystems. However, when photosystems were not fully activated under low light, the promotive effect of blue light on photosynthetic electron transport could not be fully exhibited.

To assess the downstream reaction status of PSII, the activities of key Calvin cycle enzymes and the expression levels of related genes were determined. The results revealed that blue light significantly increased the activities of Rubisco and RCA and upregulated the gene expression of key enzymes (Figure 5 and Figure 6). These findings were consistent with previous studies on tomato and eggplant, suggesting that blue light improved the maximum photosynthetic capacity of plants mainly by activating the Calvin cycle enzymes [18,47].

In this experiment, photochemical limitation remained minor throughout photoinduction, as photosynthetic electron transport reached a steady state within 5 min after light induction (Figure 3D). Biochemical limitation linked to Calvin-cycle activation dominated the early induction stage. As induction progressed, biochemical limitation declined gradually, whereas stomatal limitation rose in the mid-induction stage and lasted longer (Figure S2). Red-to-blue-light ratios altered the severity of these limitations. Monochromatic red light inhibited Calvin-cycle-related enzyme activity and gene expression (Figure 5 and Figure 6), which kept biochemical limitation at a high level over the whole induction period (Figure S2). Although monochromatic red light had low steady-state stomatal conductance (Table 1), calculated instantaneous stomatal limitation did not increase accordingly (Figure S2). These results indicated that impaired dynamic photosynthesis under monochromatic red light was mainly caused by sustained biochemical constraints rather than stomatal limitation. In summary, the dominant factor restricting photoinduction varies with plant species, fluctuating light regimes, and other environmental factors. Balancing stomatal conductance, photochemical reactions and biochemical reactions serves as an effective strategy to accelerate photoinduction, thereby reducing carbon and water loss under fluctuating light.

### 3.3. Correlation Analysis Between Key Parameters

Correlation analysis of key parameters revealed that higher chlorophyll content was positively associated with the maximum photosynthetic capacity of cucumber seedlings. However, such an association was not significant under low-light intensity (Figure 7). Consistent results were also obtained for cucumber via light-response curve measurements [46]. For steady-state photosynthetic parameters, A_f_, Gs_f_, A_f_ − A_i_ and Gs_f_ − Gs_i_ showed positive correlations with one another (Figure 7), suggesting that the total increments in A and Gs were closely associated with maximum photosynthetic capacity. In terms of dynamic photosynthetic parameters, A_f_, A_f_ − A_i_, Gs_i_, and Gs_f_ were negatively correlated with T_50%A_ and T_90%A_ (Figure 7). These relationships indicated that higher A and Gs throughout photoinduction were closely associated with faster induction kinetics. These findings agreed with previous studies reporting an association between higher Gs and faster photoinduction in tomato [57]. However, it should be noted that higher Gs would accelerate water loss via transpiration and thereby reduce water-use efficiency. Accordingly, dynamic photosynthesis might be modulated by soil water content and atmospheric moisture conditions. For instance, previous studies reported that drought and salt stresses could alter dynamic photosynthesis and plant growth in rice and tomato [58,59]. A_i_ and ETR_i_ were positively correlated with T_50%A_ (Figure 7), suggesting that higher initial photosynthetic rate and electron transport rate were associated with faster photoinduction during the early stage of light induction. T_50%Gs_, T_90%Gs_ and λ exhibited no significant correlations with other parameters, and Sl_max_ was not significantly correlated with other dynamic photosynthetic parameters. These results might be attributed to the fact that low-light pre-treatment elevated stomatal conductance, thus weakening stomatal limitation on photoinduction. Furthermore, other critical factors also affected the induction rates of A and Gs. For example, stomatal morphology, stomatal density and mesophyll conductance were reported to modulate dynamic photosynthesis in previous studies [60,61]. Rubisco activity showed a positive correlation only with ETR_f_, suggesting that the in vitro activities of key Calvin cycle enzymes and the expression levels of their encoding genes could not fully reflect their actual in vivo activities. Future studies should aim to quantify the in vivo activities of key Calvin cycle enzymes during photosynthesis via gas-exchange and biochemical approaches, so as to unravel the key limiting factors of photosynthesis.

However, it should be noted that the present experiment was conducted only with cucumber seedlings at the single-leaf scale under controlled phytotron conditions at a growth light intensity of 100 μmol·m^−2^·s^−1^. No measurements of biomass, yield, or long-term developmental performance were obtained in this study. Therefore, two key questions remain to be verified: whether the positive effects of seedling-stage red-to-blue-light treatments persist into later growth stages, and whether the optimal red-to-blue-light ratios identified here are applicable to mature cucumber plants under greenhouse or commercial production settings where environmental factors are far more complex and highly variable. Although previous work has shown that blue-light acclimation at the seedling stage can promote subsequent cucumber growth under full-sunlight conditions [62], further studies under greenhouse conditions are needed to address these questions.

## 4. Materials and Methods

### 4.1. Plant Materials and Experimental Design

This experiment was carried out in the phytotron at the Institute of Urban Agriculture, Chinese Academy of Agricultural Sciences; cucumber (*Cucumis sativus* L.) cv. ‘Jinyou 365’ seedlings were used as plant materials. After being fully soaked in distilled water for 8 h, cucumber seeds were germinated in an incubator at a temperature of 28 °C with 80% relative humidity for 20 h, and then sown in 72-hole trays (54.0 cm long × 30.0 cm wide × 5 cm deep) filled with a mixed substrate of peat, vermiculite and perlite at a volume ratio of 3:1:1 for seedling cultivation. Seedling cultivation was performed in an artificial climate chamber with the following environmental conditions: air temperature of 24 ± 3 °C, relative humidity of 60%, CO_2_ concentration of 400 ± 20 µmol·mol^−1^, light intensity (white light) of 100 μmol·m^−2^·s^−1^ with a 14 h photoperiod (7:00–21:00). When the seedlings’ cotyledons were fully unfolded but the first true leaf had not yet expanded (7 d after sowing), uniform seedlings were selected, their root substrates were carefully washed away from the roots to minimize mechanical damage, and the seedlings were transplanted into black rectangular plastic pots (NH_4_H_2_PO_4_ 0.5 mmol·L^−1^, Ca(NO_3_)_2_·4H_2_O 2.0 mmol·L^−1^, KNO_3_ 3.2 mmol·L^−1^, MgSO_4_·7H_2_O 1.0 mmol·L^−1^, with full-strength trace elements) for light treatments. Before the initiation of light treatments, seedlings were acclimated for half a day under white light to mitigate potential transplanting stress. During the treatments, cucumber seedlings were fixed with rigid foam boards and circular sponge blocks, with 20 seedlings planted per plastic pot. An air pump was used to add oxygen, and the nutrient solution was replaced every 3 days. The electrical conductivity (EC) of the nutrient solution was 2.0–2.3 mS·cm^−1^, and the pH was 6.8–7.2.

In this study, 8 treatments were set, including W (white light), R (monochromatic red light), B (monochromatic blue light), and mixed red and blue light with blue light ratios of 10%, 30%, 50%, 70% and 90% (9R1B, 7R3B, 5R5B, 3R7B and 1R9B). All light treatments, including monochromatic red light (R) and monochromatic blue light (B), as well as mixed red and blue light, were calibrated to an identical photosynthetic photon flux density (PPFD) of 100 μmol·m^−2^·s^−1^ based on photon number. Detailed spectral information for each treatment can be found in our previous study [16]. The photoperiod was 14 h·d^−1^ (7:00–21:00). The peak wavelengths of red and blue light were 660 nm and 450 nm. Other environmental conditions were the same as those used at the seedling cultivation stage. Shading cloths were placed between different treatments to prevent light interference among treatments. Heliospectra DYNA LED lamps (Heliospectra, Gothenburg, Sweden) were used as the light source. Detailed information on this type of lamp is available at ‘https://heliospectra.com/led-grow-lights/dyna/’ (accessed on 30 July 2026).

After 9 d of light treatment, the first true leaf of cucumber seedlings under all treatments had fully expanded (Figure S3). The photosynthetic pigment contents, gas-exchange parameters and chlorophyll fluorescence parameters of the first true leaf were determined. For biochemical analyses, the first true leaves of cucumber seedlings were sampled, wrapped in aluminum foil, rapidly frozen in liquid nitrogen, and then stored at −80 °C. Three independent biological replicates were used in this experiment, with at least three uniformly grown seedlings selected from each treatment for measurements.

### 4.2. Photosynthetic Pigment Contents

Photosynthetic pigment contents were measured using the 95% ethanol extraction method with little modification. The pigment contents were determined on both a leaf fresh weight basis and a leaf area basis according to the methods described in previous studies [20,46]. Briefly, five leaf disks (1.0 cm^2^) were punched from each leaf with a perforator, avoiding the main veins. The total fresh weight (FW) of these leaf disks was measured using an analytical balance (readability: 0.0001 g, model ME204E, METTLER TOLEDO, Zurich, Switzerland). Then, 5 leaf disks were immersed in 20 mL of 95% ethanol solution for 48 h in darkness. After extraction, the absorbance values of the leaf extract were determined using an ultraviolet–visible spectrophotometer (UV-1800, Shimadzu, Japan) at wavelengths of 663 nm (OD_663_), 645 nm (OD_645_) and 470 nm (OD_470_) for the quantification of chlorophyll a, chlorophyll b and carotenoids, respectively. The contents of chlorophyll a (Chl a), chlorophyll b (Chl b) and carotenoids (Car) per leaf area were calculated using the following equations:

Chl a (mg·cm^−2^) = (12.72 × OD_663_ − 2.59 × OD_645_) × V/(1000 × LA)

Chl b (mg·cm^−2^) = (22.88 × OD_645_ − 4.67 × OD_663_) × V/(1000 × LA)

Car (mg·cm^−2^) = (1000 × OD_470_ − 3.27 × Chl a − 104 × Chl b) × V/(229 × 1000 × LA)

where V was the total volume of 95% ethanol extract (20 mL), and LA was the leaf area of sampled disks (5 cm^2^). To obtain photosynthetic pigment contents per leaf fresh weight, LA in the formulas was substituted with fresh weight (FW). The ratio of chlorophyll a content to chlorophyll b content (Chl a/b) was calculated as Chl a/b = Chl a/Chl b.

### 4.3. Photosynthetic Gas-Exchange Parameters During Light Induction

The LI-6800 portable photosynthesis system equipped with a leaf chamber fluorometer (LI-COR Biosciences, Lincoln, NE, USA) was used to determine the photosynthetic gas-exchange parameters during light induction. During measurements, the air flow rate was 500 μmol·s^−1^, CO_2_ concentration was 400 μmol·mol^−1^, air relative humidity was 60%, leaf temperature was 25 °C, and fan speed was 10,000 rpm. Mixed red (80%, 635 nm) and blue (20%, 465 nm) LED light was used as the light source. We adopted the one-step light-shift method to measure photoinduction curves and calculate associated parameters to evaluate the dynamic photosynthetic capacity of cucumber seedlings, referring to previous studies on dynamic photosynthesis [22,63]. Light-response curves were determined for the first true leaf of cucumber seedlings across all treatments. The light intensity of 1500 μmol·m^−2^·s^−1^ was selected as high light that could fully activate the photosynthetic apparatus because net photosynthetic rates of all treatments reached a plateau at this intensity without significant photosynthetic inhibition (Figure S4). Before measurements, the light intensity inside the leaf chamber was set to 100 μmol·m^−2^·s^−1^. Subsequently, the first true leaf of cucumber seedlings was clamped into the leaf chamber for light acclimation. Once the net photosynthetic rate (A) and stomatal conductance (Gs) reached a steady state, an automated program was activated to measure induction curves. The program was configured as follows: data were recorded for 3 min at 100 μmol·m^−2^·s^−1^, followed by an increase in light intensity to 1500 μmol·m^−2^·s^−1^, with data recording continuing for another 27 min. The data recording interval was set to 10 s.

After measurements, the following parameters could be directly derived: initial net photosynthetic rate (A_i_) and stomatal conductance (Gs_i_) before light induction, and steady-state net photosynthetic rate (A_f_) and stomatal conductance (Gs_f_) after light induction. Intrinsic water-use efficiency (iWUE) was calculated as iWUE = A/Gs [64].

The induction state of A at different time points was calculated as follows:

$${\mathrm{IS}}_{t}\left(\%\right)=\frac{A_{t}-A_{i}}{A_{f}-A_{i}}\times 100$$

In this formula, A_t_ was the net photosynthetic rate at different time points, A_i_ was the initial net photosynthetic rate before light induction, and A_f_ was the steady-state net photosynthetic rate after light induction. According to this equation, the times to reach 50% and 90% steady-state net photosynthetic rate (T_50%A_ and T_90%A_) can be calculated [63]. The times to reach 50% and 90% steady-state stomatal conductance (T_50%Gs_ and T_90%Gs_) can be obtained similarly.

The lag time of stomatal conductance in response to light induction (λ) was calculated by the following formula:

$${\mathrm{Gs}}_{t}=\left({\mathrm{Gs}}_{f}-{\mathrm{Gs}}_{i}\right)e^{-e^{\left(\frac{\lambda -t}{k}+1\right)}}+{\mathrm{Gs}}_{i}$$

In this formula, Gs_t_ was the stomatal conductance at different time points, Gs_f_ was the steady-state stomatal conductance after light induction, Gs_i_ was the initial stomatal conductance before light induction, λ was the lag time for stomatal conductance response, and k was a time constant [30]. According to this formula, the data were fitted using Origin 2021 (OriginLab Corp., Northampton, MA, USA); Gs_t_ was set as the dependent variable (y), t was the independent variable (x), and (Gs_f_ − Gs_i_) and Gs_i_ were constants. λ and k were obtained by fitting Gs_t_ and t [16]. Then, the maximum increase rate of stomatal conductance (Sl_max_) was calculated using the following formula:

$${\mathrm{Sl}}_{\max}=\frac{{\mathrm{Gs}}_{f}-{\mathrm{Gs}}_{i}}{k\times e}$$

To calculate instantaneous stomatal limitation (LS) and instantaneous biochemical limitation (LB) during light induction, the non-stomatal-limited net photosynthetic rate (A*) was calculated by the following formula:

$$A^{*}=\frac{\left(A_{t}+R_{L})({\mathrm{Ci}}_{f}-\Gamma^{*}\right)}{{\mathrm{Ci}}_{t}-\Gamma^{*}}-R_{L}$$

In this formula, A_t_ was the net photosynthetic rate at different time points, R_L_ was the light-adapted dark respiration rate, Ci_f_ was the steady-state intercellular carbon dioxide concentration after light induction, Γ* was the carbon dioxide compensation point at the chloroplast carboxylation site, and Ci_t_ was the intercellular carbon dioxide concentration at different time points [22]. The values of Γ* and R_L_ were determined in our previous study [16].

Instantaneous stomatal limitation (LS) and biochemical limitation (LB) during light induction were calculated using the following formulas:

$$\mathrm{LS}=\frac{A^{*}-A_{t}}{A_{f}+R_{L}}$$

$$\mathrm{LB}=\frac{A_{f}-A^{*}}{A_{f}+R_{L}}$$

### 4.4. Chlorophyll Fluorescence Parameters During Light Induction

Chlorophyll fluorescence parameters were determined simultaneously with photosynthetic gas-exchange measurements using a LI-6800 portable photosynthesis system equipped with a fluorescence leaf chamber (LI-COR Biosciences, Lincoln, NE, USA). Cucumber seedlings subjected to overnight dark adaptation were used to measure the minimum fluorescence (Fo) and the maximum fluorescence (Fm) of photosystem II (PSII). For saturation pulse settings, a rectangular flash was applied, with a red target intensity of 12,000 μmol·m^−2^·s^−1^, a pulse duration of 1000 ms, an output rate of 100 Hz, and a margin of 5 points. Following dark-adapted fluorescence measurements, the photochemical light intensity was set to 100 μmol·m^−2^·s^−1^ for light acclimation. After A and Gs stabilized, the same automated program was used to record photosynthetic gas exchange and chlorophyll fluorescence parameters simultaneously. For light-adapted fluorescence measurements, a Multi-Phase saturation flash was used to avoid high-light damage to cucumber leaves. Chlorophyll fluorescence parameters were recorded at 2 min intervals.

After measurement, the following parameters were calculated. Maximum photochemical efficiency of PSII under light adaptation (Fv’/Fm’) was calculated as Fv’/Fm’ = (Fm’ − Fo’)/Fm’. The photochemical quenching coefficient of PSII (qP) was calculated as qP = (Fm’ − Fs)/(Fm’ − Fo’). The actual photochemical quantum yield of PSII (Y(II)) was calculated as Y(II) = (Fm’ − Fs)/Fm’. Relative electron transport rate of PSII (ETR) was calculated as ETR = Y(II)·PPFD·0.84·0.5, where PPFD is the photosynthetic photon flux density, 0.84 is the leaf absorption coefficient, and 0.5 represents the fraction of light allocated to PSII. Quantum yield of regulated non-photochemical energy dissipation of PSII (Y(NPQ)) was calculated as Y(NPQ) = Fs/Fm’ − Fs/Fm. Quantum yield of non-regulated non-photochemical energy dissipation of PSII (Y(NO)) was calculated as Y(NO) = Fs/Fm. Here, Fm was dark-adapted maximum fluorescence, Fm’ was light-adapted maximum fluorescence, Fs was light-adapted steady-state fluorescence, and Fo’ was light-adapted minimum fluorescence [65].

### 4.5. Activities and Gene Expression Levels of Calvin Cycle-Related Enzymes

The activities of ribulose-1,5-bisphosphate carboxylase/oxygenase (Rubisco) and Rubisco activase (RCA) were determined using ELISA kits (MM-0603O2 and MM-0602O2, Jiangsu Meimian Industrial Co., Ltd., Yancheng, China).

The gene expression levels of Calvin cycle enzymes were determined by quantitative real-time PCR (qRT-PCR). Firstly, total RNA was extracted from cucumber leaves using an RNA Extraction Kit (DP452, Tiangen Biotech Co., Ltd., Beijing, China). After extraction, RNA concentration and purity were quantified using a NanoDrop One spectrophotometer (Thermo Fisher Scientific, Waltham, MA, USA). Subsequently, 1.5 μg of total RNA was subjected to reverse transcription with a FastKing First-Strand cDNA Synthesis Kit (KR116, Tiangen Biotech Co., Ltd., Beijing, China). The resulting cDNA was used for subsequent qRT-PCR analysis. The Talent Fluorescence quantitative detection Kit (SYBR Green) (FP209, Tiangen Biotech Co., Ltd., Beijing, China) and QuantStudio 5 instrument (Thermo Fisher Scientific, Waltham, MA, USA) were used for qRT-PCR analysis. The total reaction volume was 20 μL, with a final cDNA template concentration of 1 ng·μL^−1^. The PCR procedure consisted of an initial pre-denaturation at 95 °C for 3 min, followed by 40 cycles of 95 °C for 5 s, 60 °C for 10 s, and 72 °C for 15 s. Fluorescence signals were collected at 72 °C. Relative gene expression levels were calculated using the 2^−ΔΔCT^ method. Gene-specific primers (Table S1) were synthesized by Shanghai Bioengineering Co., Ltd., Shanghai, China, and their sequences were consistent with those reported in a previous study [66].

### 4.6. Statistics

The mean values and standard deviations (SD) were calculated using Microsoft Excel 2016 (Microsoft Corporation, Redmond, WA, USA). Data normality and homogeneity of variance were examined using SPSS 16.0 (IBM, New York, NY, USA). Subsequently, one-way analysis of variance (ANOVA) was performed, followed by Tukey’s HSD multiple comparison test to identify significant differences among treatments at *p* < 0.05. Graphs were generated with Origin 2021 (OriginLab Corp., Northampton, MA, USA). Pearson correlation analysis with a two-tailed test was conducted using the Correlation Plot App (v1.31) in Origin 2021 to evaluate the relationships between the key parameters.

## 5. Conclusions

Compared with red light, blue light under mixed red–blue light can significantly increase photosynthetic pigment contents, the maximum photosynthetic capacity, electron transport capacity, photoinduction rate, and the activities and gene expression of key Calvin cycle enzymes. However, the promotive effects of blue light on photosynthetic capacity under low-light intensity are negligible. Higher chlorophyll contents are associated with the greater maximum photosynthetic capacity of cucumber seedlings but show no significant association with photoinduction. Higher A and Gs during light-induction transitions are associated with faster photoinduction. These results indicate that under low-light intensity (100 μmol·m^−2^·s^−1^), both the maximum and dynamic photosynthesis of cucumber seedling leaves can be improved by optimizing the blue-light ratio, but the maximum and dynamic photosynthetic capacity require different optimal blue-light ratios.

## Figures and Tables

**Table 1:** Steady-state photosynthetic gas-exchange parameters during light induction.

Treatments	A_i_	A_f_	Gs_i_	Gs_f_	A_f_ − A_i_	Gs_f_ − Gs_i_
	(μmol·m^−2^·s^−1^)	(μmol·m^−2^·s^−1^)	(mol·m^−2^·s^−1^)	(mol·m^−2^·s^−1^)	(μmol·m^−2^·s^−1^)	(mol·m^−2^·s^−1^)
W	3.93 ± 0.42 ab	13.92 ± 1.25 c	0.18 ± 0.035 abc	0.31 ± 0.039 d	9.99 ± 1.05 bc	0.13 ± 0.008 c
R	3.10 ± 0.40 b	6.75 ± 1.09 d	0.06 ± 0.001 d	0.11 ± 0.004 f	3.65 ± 0.97 d	0.05 ± 0.013 c
9R1B	4.46 ± 0.69 a	13.30 ± 0.89 c	0.11 ± 0.009 cd	0.36 ± 0.022 d	8.84 ± 0.41 c	0.25 ± 0.016 b
7R3B	4.97 ± 1.01 a	16.75 ± 1.42 b	0.25 ± 0.039 a	0.53 ± 0.017 b	11.78 ± 1.99 b	0.28 ± 0.035 b
5R5B	4.39 ± 0.73 a	18.82 ± 1.94 ab	0.21 ± 0.076 ab	0.51 ± 0.067 b	14.43 ± 1.32 a	0.29 ± 0.115 b
3R7B	4.30 ± 1.29 ab	19.90 ± 1.75 a	0.21 ± 0.084 ab	0.61 ± 0.041 a	15.6 ± 0.84 a	0.40 ± 0.081 a
1R9B	4.80 ± 0.48 a	18.50 ± 0.88 ab	0.17 ± 0.047 abc	0.44 ± 0.054 c	13.70 ± 0.70 a	0.27 ± 0.071 b
B	4.57 ± 0.87 a	13.48 ± 2.05 c	0.14 ± 0.001 bcd	0.19 ± 0.001 e	8.91 ± 1.91 c	0.05 ± 0.010 c

Initial net photosynthetic rate before light induction (A_i_), steady-state net photosynthetic rate after light induction (A_f_), initial stomatal conductance before light induction (Gs_i_), steady-state stomatal conductance after light induction (Gs_f_), total increment in net photosynthetic rate during light induction (A_f_ − A_i_), total increment in stomatal conductance during light induction (Gs_f_ − Gs_i_). Data in this table are presented as means ± SD (*n* = 3). Different letters indicate significant differences among treatments (*p* < 0.05).

**Table 2:** Dynamic photosynthetic gas-exchange parameters during light induction.

Treatments	T_50%A_	T_90%A_	T_50%Gs_	T_90%Gs_	λ	Sl_max_
	(s)	(s)	(s)	(s)	(min)	(mol·m^−2^·s^−1^)
W	263 ± 15 bc	983 ± 167 a	977 ± 47 a	1273 ± 21 a	7.36 ± 1.15 b	0.0147 ± 0.0015 c
R	443 ± 23 a	1103 ± 150 a	733 ± 46 bc	1127 ± 266 a	4.85 ± 0.12 c	0.0030 ± 0.0003 d
9R1B	327 ± 51 b	900 ± 218 a	823 ± 150 abc	770 ± 192 b	6.29 ± 0.76 b	0.0212 ± 0.0015 bc
7R3B	237 ± 6 c	477 ± 67 b	767 ± 51 bc	1277 ± 64 a	2.86 ± 0.72 d	0.0177 ± 0.0008 bc
5R5B	277 ± 55 bc	563 ± 157 b	653 ± 31 c	1203 ± 129 a	2.71 ± 0.46 d	0.0210 ± 0.0011 bc
3R7B	253 ± 6 c	483 ± 110 b	813 ± 51 abc	1240 ± 52 a	1.91 ± 0.36 d	0.0180 ± 0.0039 bc
1R9B	303 ± 68 bc	880 ± 165 a	893 ± 72 ab	1233 ± 50 a	3.16 ± 0.68 d	0.0258 ± 0.0044 a
B	263 ± 12 bc	947 ± 300 a	900 ± 173 ab	1243 ± 12 a	12.82 ± 0.98 a	0.0188 ± 0.0018 bc

Time to reach 50% steady-state net photosynthetic rate (T_50%A_), time to reach 90% steady-state net photosynthetic rate (T_90%A_), time to reach 50% steady-state stomatal conductance (T_50%Gs_), time to reach 90% steady-state stomatal conductance (T_90%Gs_), lag time for stomatal conductance response (λ), the maximum increase rate of stomatal conductance (Sl_max_). Data in this table are presented as means ± SD (*n* = 3). Different letters indicate significant differences among treatments (*p* < 0.05).

## Data Availability

Data are contained within the article and Supplementary Materials.

## Supplementary Materials

The following supporting information can be downloaded at: https://www.mdpi.com/article/10.3390/plants15193021/s1. Figure S1: Leaf photosynthetic pigment contents per leaf fresh weight (FW); Figure S2: Limitation analysis during light induction; Figure S3: Morphological characteristics of cucumber seedlings under different treatments; Figure S4: Light-response curves of the first true leaf of cucumber seedlings under different treatments; Table S1: Gene-specific primers for qRT-PCR analysis.
