# Supplementary material for: Effects of Red and Blue Light on Steady-State and Dynamic Photosynthesis of Cucumber Seedlings
Source: Plants (Basel). 2026 Oct 2;15(19):3021. doi: 10.3390/plants15193021 (PMC13667014; doi:10.3390/plants15193021)
Supplement: Supplementary file 1 [file plants-15-03021-s001.zip › plants-4574809-supplementary.pdf]

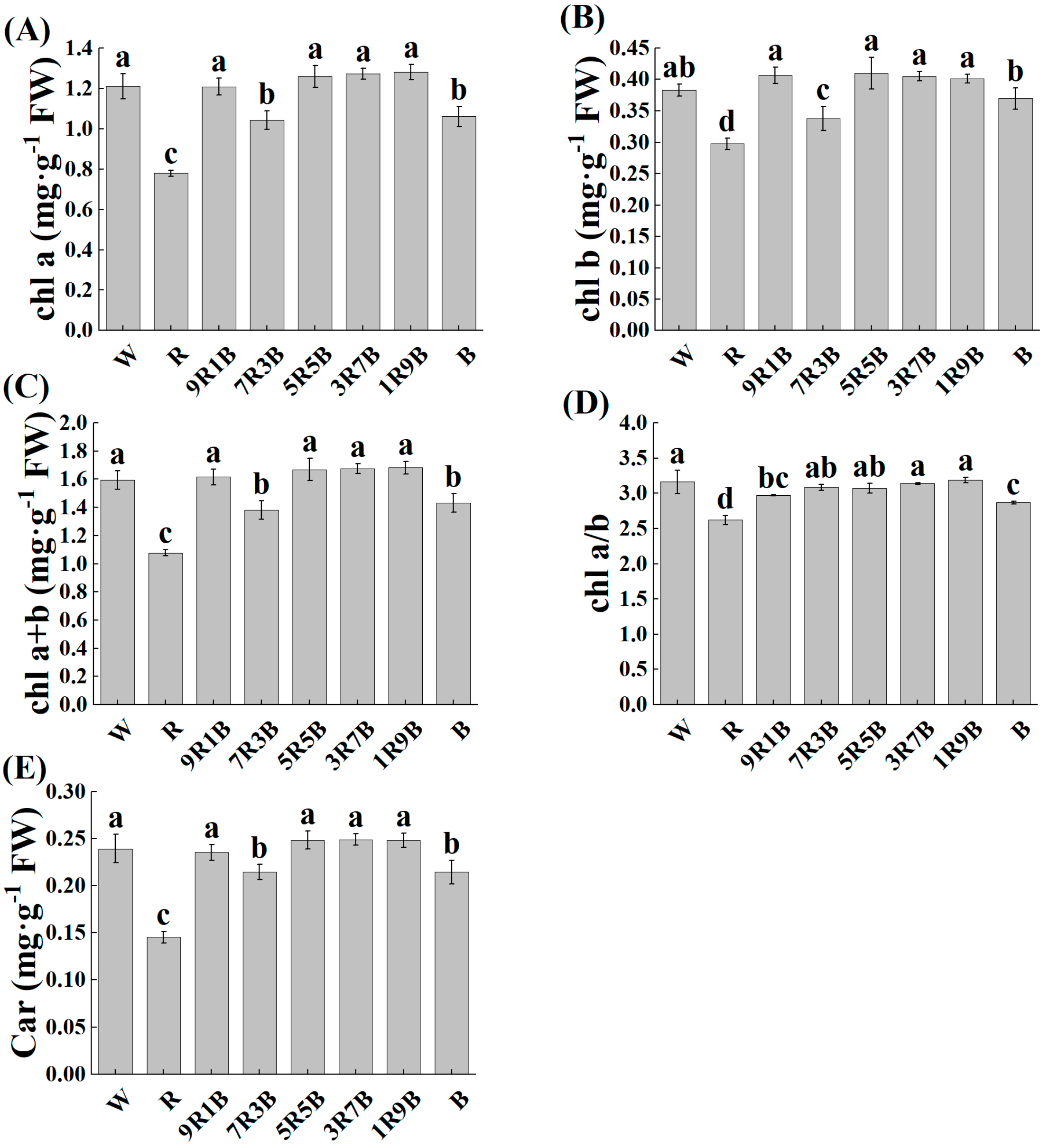

Figure S1. Leaf photosynthetic pigment contents per leaf fresh weight (FW). (A): chlorophyll a content per leaf fresh weight (Chl a), (B): chlorophyll b content per leaf fresh weight (Chl b), (C): total chlorophyll content per leaf fresh weight (Chl a+b), (D): ratio of chlorophyll a content to chlorophyll b content (Chl a/b), (E): carotenoid content per leaf fresh weight (Car). White light (W), monochromatic red light (R), 90% red and 10% blue mixed light (9R1B), 70% red and 30% blue mixed light (7R3B), 50% red and 50% blue mixed light (5R5B), 30% red and 70% blue mixed light (3R7B), 10% red and 90% blue mixed light (1R9B), and monochromatic blue light (B). Different letters indicate significant differences among treatments ($p < 0.05$). The same for the following figures.

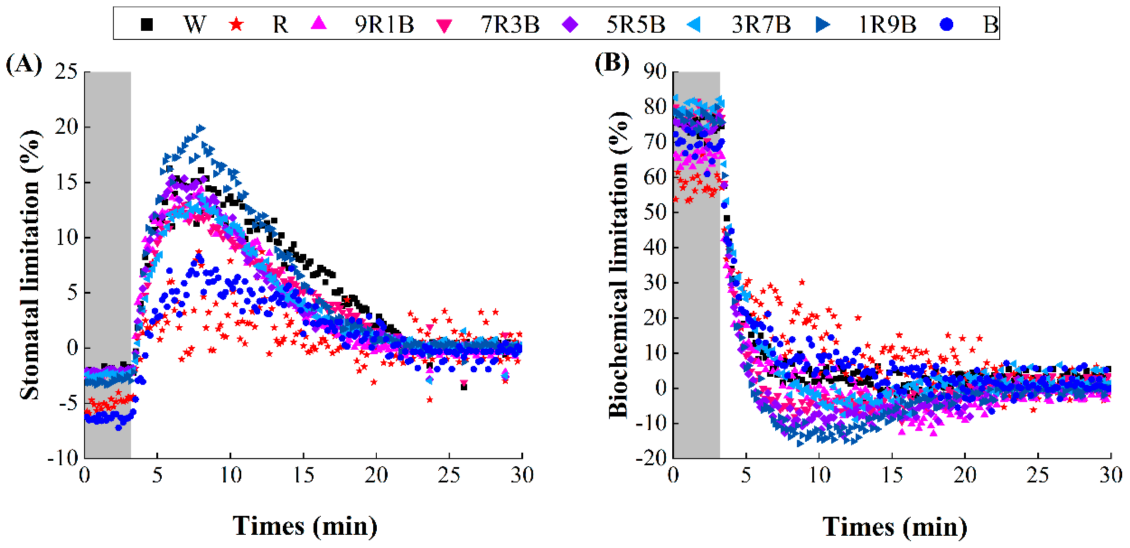

Figure S2. Limitation analysis during light induction. (A): stomatal limitation during light induction, (B): biochemical limitation during light induction. The gray area represents a light intensity of 100 μmol·m⁻²·s⁻¹, and the white area represents a light intensity of 1500 μmol·m⁻²·s⁻¹. Light intensity was increased at 3 min.

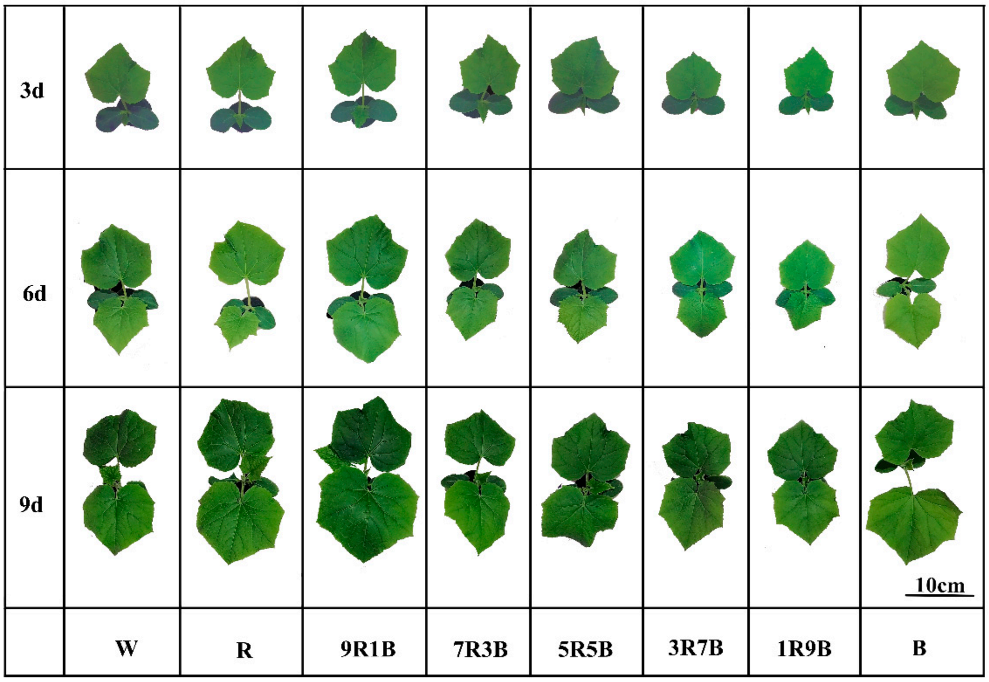

Figure S3. Morphological characteristics of cucumber seedlings under different treatments.

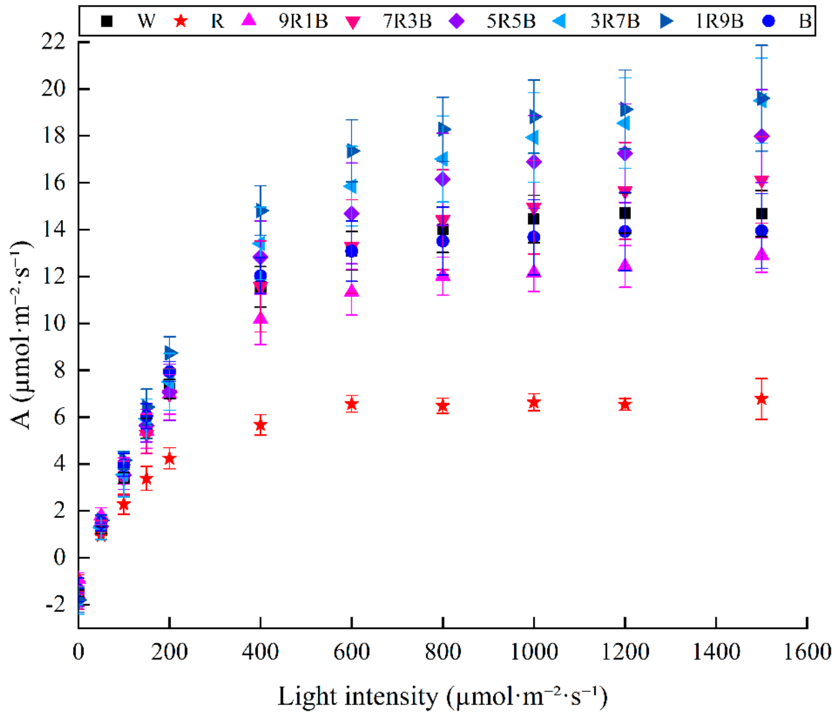

Figure S4. Light response curves of the first true leaf of cucumber seedlings under different treatments.

Table S1. Gene-specific primers for qRT-PCR analysis.

Gene name	Gene ID	Primer sequences (5'-3')
CsTUB	AJ715498	F: GCCACTACACCGTTGGAAAGGAAAT<br>R: AGGAGGGAGCCGAGACCAGAAC
CsrbcL	EF208123.1	F: TACTGATATCTTGGCAGCATTCCG<br>R: AAGATTCAGCAGCTACAGCGGC
CsrbcS	NM_001405108.1	F: ATGGCTTCATCCATTCTCTCATCC<br>R: CCAGTGAATGGTGCTACCATGCTA
CsRca	NM_001280726.1	F: GAATATGGCAACATGCTCGTCATG<br>R: TCCAAGAGCAGCTTCGTTCAGAT
CsFBPA	XM_004150088.3	F: GCAGCAACCAGATCAGAGAGCGTT<br>R: GGAAGGAGGTTGAACAGAGTGTTGA
CsFBPase	XM_004147851.3	F: AATCTCTCGTTCTCTCTCTCGCCTC<br>R: CGCTATGCCTTCTATTTCCACGG
CsTPI	XM_004146969.3	F: CTCTCTTTCACAACGTCCACTCACA<br>R: ACCAACGAAGAACTTGCCGGAG
CsSBPase	NM_001280729.1	F: CAGTGTCCTCCTCATACTTGGGTTG<br>R: CTGGGAAGAAAGATTGGGGAGAAA
CsRupe	XM_004137370.3	F: TCCCAAGTCAGTGGGTTTATCGGAG<br>R: AACCTTCTCCTCGAAACGGTAAGAG
CsPRK	XM_004144364.3	F: ATCCACACCCTCATTCATTTCTCC<br>R: GCAGTTGAGGGAGTGAAGAAGAAGA

Cucumber Rubisco large subunit gene (*CsrbcL*), cucumber Rubisco small subunit gene (*CsrbcS*), cucumber Rubisco activase gene (*CsRca*), cucumber fructose-1,6-bisphosphate aldolase gene (*CsFBPA*), cucumber fructose-1,6-bisphosphatase gene (*CsFBPase*), cucumber triose-phosphate isomerase gene (*CsTPI*), cucumber sedoheptulose-1,7-bisphosphatase gene (*CsSBPase*), cucumber ribulose-5-phosphate epimerase gene (*CsRupe*), and cucumber phosphoribulokinase gene (*CsPRK*). The cucumber β-tubulin gene (*CsTUB*) was used as the reference gene.
